# DNA methyltransferase activity in the basolateral amygdala is critical for reconsolidation of a heroin reward memory

**DOI:** 10.3389/fnmol.2022.1002139

**Published:** 2022-09-13

**Authors:** Shuyi Qian, Cuijie Shi, Shihao Huang, Chang Yang, Yixiao Luo

**Affiliations:** ^1^Department of Nephrology and Laboratory of Kidney Disease, Hunan Provincial People’s Hospital, Hunan Normal University, Changsha, China; ^2^Hunan Province People’s Hospital, The First-Affiliated Hospital of Hunan Normal University, Changsha, China; ^3^National Institute on Drug Dependence, Beijing Key Laboratory of Drug Dependence, Peking University, Beijing, China

**Keywords:** addiction, heroin, amygdala, reconsolidation, DNMT, self-administration

## Abstract

The persistence of drug memory contributes to relapse to drug seeking. The association between repeated drug exposure and drug-related cues leads to cravings triggered by drug-paired cues. The erasure of drug memories has been considered a promising way to inhibit cravings and prevent relapse. The re-exposure to drug-related cues destabilizes well-consolidated drug memories, during which a *de novo* protein synthesis-dependent process termed “reconsolidation” occurs to restabilize the reactivated drug memory. Disrupting reconsolidation of drug memories leads to the attenuation of drug-seeking behavior in both animal models and people with addictions. Additionally, epigenetic mechanisms regulated by DNA methyltransferase (DNMT) are involved in the reconsolidation of fear and cocaine reward memory. In the present study, we investigated the role of DNMT in the reconsolidation of heroin reward memory. In the heroin self-administration model in rats, we tested the effects of DNMT inhibition during the reconsolidation process on cue-induced reinstatement, heroin-priming-induced reinstatement, and spontaneous recovery of heroin-seeking behavior. We found that the bilateral infusion of 5-azacytidine (5-AZA) inhibiting DNMT into the basolateral amygdala (BLA) immediately after heroin reward memory retrieval, but not delayed 6 h after retrieval or without retrieval, decreased subsequent cue-induced and heroin-priming-induced reinstatement of heroin-seeking behavior. These findings demonstrate that inhibiting the activity of DNMT in BLA during the reconsolidation of heroin reward memory attenuates heroin-seeking behavior, which may provide a potential strategy for the therapeutic of heroin addiction.

## Introduction

A major challenge in treating heroin addiction is relapse and is closely related to the persistence of drug reward memories (Kalivas and Volkow, 2005; Robbins et al., 2008; Torregrossa et al., 2011; Wang et al., 2012; Preller et al., 2013; Chen et al., 2021a; Ewing et al., 2021; Douton et al., 2022; Xie et al., 2022). Drug memories are maladaptive memories that usurp normal memory, leading to craving and relapse (Hyman, 2005; Hyman et al., 2006; Böning, 2009; Alvandi et al., 2017; Stern et al., 2018; Liu et al., 2019; Chen et al., 2021b). Previous studies have reported that memory traces become labile after reactivation and are re-stabilized through a process termed “reconsolidation” (Nader, 2003; Nader and Einarsson, 2010; Alberini and Ledoux, 2013; Tronson and Taylor, 2013). Both human and animal studies demonstrate that drug-seeking behavior is impaired by the pharmacological or non-pharmacological interference in the reconsolidation of drug reward memories (Lee et al., 2005; Xue et al., 2012; Lin et al., 2014; Chen et al., 2021a; Zhang et al., 2021; Xie et al., 2022). Therefore, clarifying the underlying mechanism in the reconsolidation of drug memories will help determine the pharmacological target for the prevention of relapse.

The basolateral amygdala (BLA), a subregion of the amygdala, is closely implicated in learning, memory, and emotional behavior (Davis, 1992; Maren, 2003; See et al., 2003). Substantial evidence indicates that the BLA is a critical brain region involved in the reconsolidation of drug-related associative memories (Hellemans et al., 2006; Wells et al., 2013; Jian et al., 2014; Higginbotham et al., 2021; Ritchie et al., 2021). Two important aspects of memory reconsolidation have been pointed out. One is that *de novo* protein synthesis is required during the reconsolidation process and the other is that reconsolidation occurred within a 6 h time window (Nader et al., 2000). Therefore, previous studies have demonstrated that disrupting protein synthesis for reconsolidation in the BLA reduces relapse of both fear and addiction memory (Fuchs et al., 2009; Wells et al., 2011; Si et al., 2012; Arguello et al., 2014; Ratano et al., 2014). Moreover, the abovementioned effects would not be observed if the intervention occurs out the time window of the reconsolidation or without the reconsolidation process (Chen et al., 2021a; Zhang et al., 2021). In short, these studies indicated that the BLA plays a critical role in the reconsolidation of heroin reward memory.

DNA methyltransferase (DNMT), widely expressed in the nervous system of mammalians, is an enzyme catalyzing DNA methylation that is critical for the formation of amygdala-dependent memory and the maintenance of long-term memory (Feng et al., 2005; Miller and Sweatt, 2007; Day and Sweatt, 2010; Sultan and Day, 2011). In the previous study, infusing the nucleoside analog 5-azacytidine (5-AZA) and DNMT inhibitor RG108 into the lateral amygdala (LA) significantly impaired the reconsolidation of fear memory (Maddox and Schafe, 2011). Our previous study found that a bilateral intra-BLA infusion of the 5-AZA after reactivation decreased subsequent cocaine-seeking behavior, indicating that the activity of DNMT in the BLA is crucial for the reconsolidation of cocaine-associated memory (Shi et al., 2015). However, whether DNMT plays a role in the reconsolidation of heroin reward memory is still unknown (Lüscher and Ungless, 2006).

In this study, we investigated the effect of intra-BLA DNMT inhibition during reconsolidation on the subsequent heroin-seeking behavior of heroin reward memory. We found that a bilateral infusion of the 5-azacytidine (5-AZA) into the basolateral amygdala (BLA) to inhibit the activity of DNMT immediately after heroin reward memory retrieval, but no longer than 6 h after retrieval or with a 5-AZA infusion without retrieval, decreased subsequent cue-induced and heroin-priming-induced reinstatement of heroin-seeking behavior.

## Materials and methods

### Subjects

The male Sprague Dawley rats weighed 280–300 g on arrival and were placed in a climate-controlled environment with a constant 22 ± 2°C temperature and 60% humidity. Food and water were freely accessible to the rats and they were under a 12-h light/dark cycle. Leading up to the surgeries, the operator handled the rats for 3 min/d for 5 day so that they would be more accustomed to the operator. The current study and all animal procedures were performed following the Guide of Hunan Province for the Care and Use of Laboratory Animals. The experiments were approved by the Local Committee on Animal Care and Use and Protection of the Hunan Normal University. The Dark phase was the time all the experiments were performed.

### Surgery

A sodium pentobarbital anesthesia (60 mg/kg, intraperitoneally) was administered to the rats (300–320 before surgery) using catheters inserted into the right jugular vein and terminating at the opening of the right atrium (Lu et al., 2005). The guide cannulae (23 gauge; Plastics One, Roanoke, VA, United States) was implanted bilaterally 1 mm above the BLA. The coordinates of the BLA are as follows (Wu et al., 2011): anterior/posterior: –2.8 mm, medial/lateral: ±5.0 mm from bregma, and dorsal/ventral: –8.5 mm from the surface of the skull. Every 2 days 0.1 ml heparinized saline (30 USP heparin/saline; Hospira) was infused through a patent catheter. As soon as the rats returned from surgery, they were housed individually and had free access to food and water. They recovered for 5–7 days before the start of the experiment.

### Behavioral procedures

#### Heroin SA training

As reported by Xue et al. (2012) the heroin SA training method and conditions were established with slight modifications. In the chambers (AniLab Software & Instruments, Ningbo, China), there were two nose poke operandi positioned 9 cm above the floor. The active nosepoke lead to an intravenous heroin infusion following a compound 5-s tone-light cue (conditioned stimulates, CS), while the inactive nosepoke had no consequence.

A 10-day training program was conducted to train the rats to self-administer heroin (0.05 mg/kg/infusion) in three 1-h training processes, separated by 5 min breaks. During training, the fixed-ratio 1 reinforcement schedule was implemented at the beginning of each dark cycle. There was a 40-s timeout period following every infusion. The house light was on when each session began. During the training sessions, the rats were deprived of food. To protect the rats from an overdose, the number of heroin infusions was limited to 20 per hour (Xue et al., 2012; Luo et al., 2015). The heroin SA paradigm was performed in all four experiments.

#### Nose poke extinction

After the SA training, rats received a 9-day nosepoke extinction training for 3 h per day with no illumination or any stimulus in the original training chamber (experiments 1–4). A nosepoke in either operandum would result in no consequences (i.e., no heroin infusion and no tone/light cue).

#### Reactivation of heroin reward memory

After 24 h, following the last nosepoke extinction (experiments 1, 2, 4), the rats were subjected to conditioned stimulus in the original training chamber for 15 min to reactivate heroin reward memory. The retrieval conditions were similar to the heroin SA training, but a nosepoke in the active operandum caused no heroin infusions.

#### 5-AZA treatment

In experiments 1 and 2, immediately after the reactivation session, the rats received bilateral infusions of the 5-AZA (1 μg/side at 0.25 μl/min for 2 min; Sigma-Aldrich) intra-BLA to inhibit the activity of DNMT. The syringe pump used the 10 μl Hamilton syringes. The syringes were linked to the infusion cannula (28 gauge; Plastics One) by the polyethylene tubing, while the controls had an equal volume infusion of vehicle (0.5% DMSO). The syringes were kept at the injection site for at least 2 min after completing the injection and then slowly withdrawn. In experiment 3, the rats received an infusion of the 5-AZA or vehicle with no light/tone stimuli reactivation. Finally, in experiment 4, the rats received infused 5-AZA or the vehicle 6 h after retrieval. The rats were placed in their home cage after infusion manipulation.

#### Cue extinction

Daily cue extinction was performed on the rats in experiments 1, 3, and 4 for 3 h. The conditions were the same as the heroin SA session but without heroin infusions following the tone/light cue.

#### Cue-induced reinstatement test (experiments 1–4)

This test was carried out on the rats 24 h after the 5-AZA or the vehicle intra-BLA infusion. The testing conditions were the same as the heroin SA session except that the active nosepoke did not have any tone-light cues, nor was it reinforced with heroin. The number of nosepokes was recorded for 1 h and the houselight was on for the whole session.

#### Heroin-induced reinstatement test (experiments 1, 3, 4)

Heroin (0.25 mg/kg, s.c.) was systematically injected into the rats for 5 min before the reinstatement test. The conditions of the test were the same as the SA training except that a nosepoke in the active operandum led to the delivery of a cue (tone/light) but not the heroin. A reinstatement test was performed for 1 h and the number of active nosepokes was recorded.

#### Spontaneous recovery test (experiment 2)

After withdrawal (28 days), the number of active nosepokes was recorded for 1 h in the spontaneous recovery test with the same conditions as in the reactivation.

### Specific experiments

#### Experiment 1

The role of immediate post-reactivation 5-AZA treatment intra-BLA on subsequent cue-induced and heroin-priming-induced reinstatement of heroin-seeking behavior.

**FIGURE 1:**
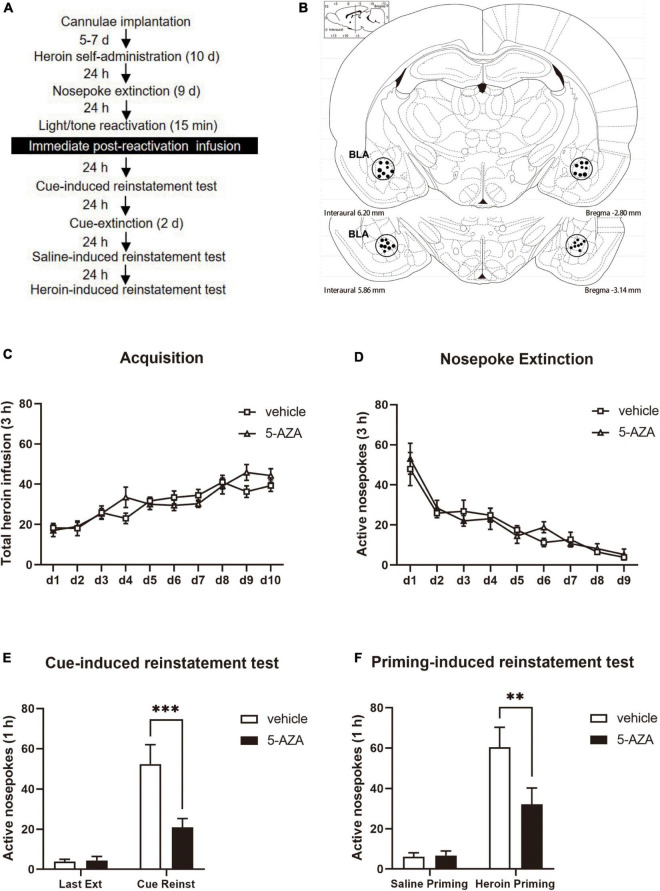
Immediate post-reactivation 5-AZA treatment intra-BLA reduces subsequent cue-induced and heroin-priming reinstatement of heroin-seeking behavior. **(A)** Schematic representation of the experimental procedure. **(B)** The regions of the representative cannula placements in the basolateral amygdala (BLA: –2.8 mm from bregma) as shown in the rostral faces of each coronal section. **(C)** Total number of heroin infusions across acquisition of heroin self-administration sessions. **(D)** Total number of active nosepoke responses across nosepoke response extinction sessions. **(E)** Active nosepoke responses during the last extinction session and the cue-induced reinstatement test. **(F)** Active nosepoke responses during the saline- or heroin- priming reinstatement test. *n* = 10 rats per group. Data are means ± SEM, ^**^p < 0.01, ^***^p < 0.001, compared with the vehicle group. Ext, extinction; Reinst, reinstatement.

The rats were subjected to the heroin SA training for 10 days in 1 h sessions, 3 times per day. They were then trained for 9 days of daily nosepoke extinction in the original chamber. After 24 h following the last nosepoke extinction, the rats were reactivated for 15 min with heroin reward cues in the training context. After reactivation, the rats were divided into two groups: (1) infusing 5-AZA intra-BLA bilaterally 1 μg in 0.5 μl/side (5-AZA group); (2) infusing vehicle intra-BLA bilaterally 0.5 μl/side (vehicle group). The rats received a bilateral infusion of either 5-AZA or vehicle intra-BLA immediately after the reactivation. After 24 h, we tested the heroin-seeking behavior of cue-induced reinstatement in rats. A priming-induced reinstatement test was conducted 2 days after the cue extinction session (see Figure 1A).

#### Experiment 2

The long-term role of immediate post-reactivation 5-AZA treatment intra-BLA on cue-induced reinstatement and spontaneous recovery.

**FIGURE 2:**
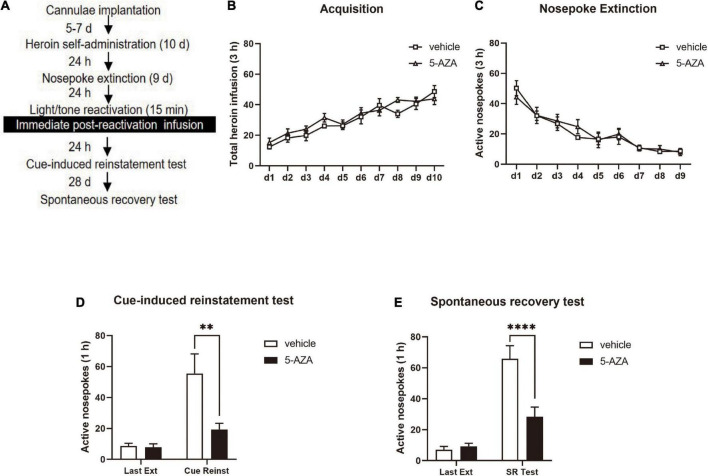
Immediate post-reactivation 5-AZA treatment intra-BLA reduces subsequent cue-induced heroin seeking and the spontaneous recovery of heroin seeking behavior. **(A)** Schematic representation of the experimental procedure. **(B)** Total number of heroin infusions across acquisition of heroin self-administration sessions. **(C)** Total number of active nosepoke responses across nosepoke response extinction sessions. **(D)** Active nosepoke responses during the last extinction session and the cue-induced reinstatement test. **(E)** Active nosepoke responses during the last extinction session and spontaneous recovery test. *n* = 8 rats per group. Data are means ± SEM, ^**^p < 0.01, ^****^p < 0.0001, compared with the vehicle group. Ext, extinction; Reinst, reinstatement; SR, spontaneous recovery.

For the heroin self-administration session and nosepoke extinction session, the conditions and tone/light reactivation in Experiment 2 were the same as in Experiment 1. The rats were subjected to the 5-AZA treatment of 1 μg in 0.5 μl/side and the controls received the vehicle immediately after a 15-min tone/light reactivation. The cue-induced reinstatement test was performed 24 h later to assess the heroin-seeking behavior. After 28 days of withdrawal, the spontaneous recovery was tested (Figure 2A).

#### Experiments 3

The role of immediate post-reactivation 5-AZA infusion intra-BLA on subsequent cue-induced and heroin-priming-induced reinstatement of heroin-seeking behavior without reactivation.

**FIGURE 3:**
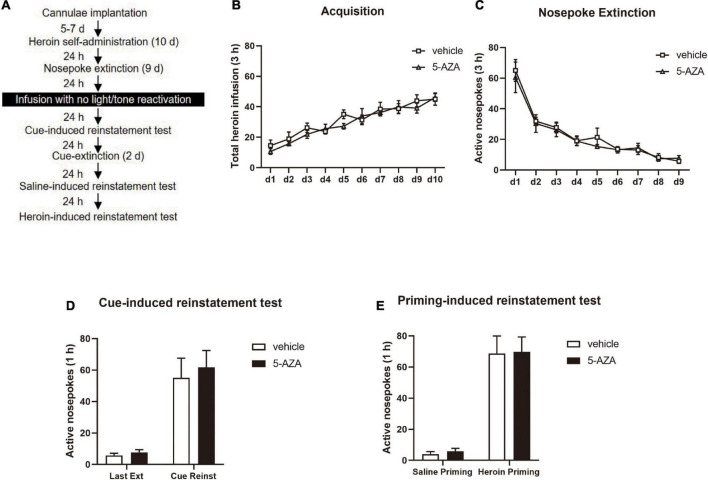
5-AZA infusion intra-BLA without reactivation has no effect on subsequent cue-induced and heroin-priming reinstatement of heroin-seeking behavior. **(A)** Schematic representation of the experimental procedure. **(B)** Total number of heroin infusions across acquisition of heroin self-administration sessions. **(C)** Total number of active nosepoke responses across nosepoke response extinction sessions. **(D)** Active nosepoke responses during the last extinction session and the cue-induced reinstatement test. **(E)** Active nosepoke responses during the saline- or heroin-priming reinstatement test. *n* = 8 rats per group. Data are means ± SEM. Ext, extinction; Reinst, reinstatement.

During Experiment 3, the experimental protocol was similar to Experiment 1, except that the rats were subjected to the infusion of 5-AZA or vehicle intra-BLA bilaterally without reactivation (see Figure 3A).

#### Experiment 4

The role of delayed 5-AZA treatment intra-BLA post-reactivation on subsequent cue- and heroin-priming-induced reinstatement of heroin-seeking behavior.

**FIGURE 4:**
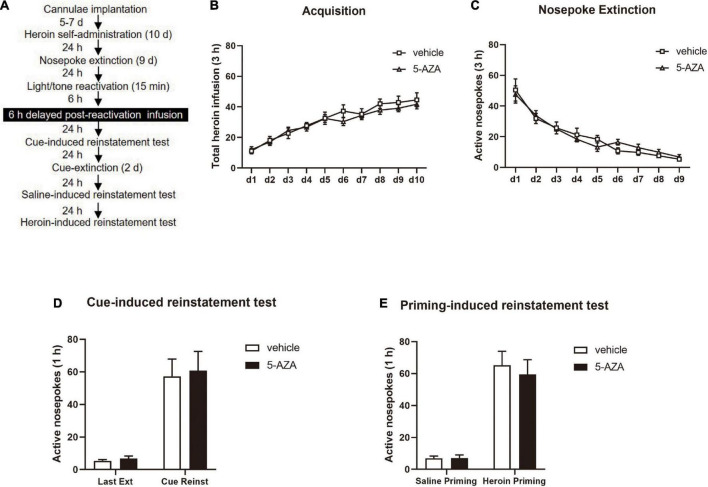
Delayed 5-AZA treatment intra-BLA following reactivation has no effect on subsequent cue-induced and heroin-priming reinstatement of heroin-seeking behavior. **(A)** Schematic representation of the experimental procedure. **(B)** Total number of heroin infusions during acquisition of heroin self-administration sessions. **(C)** Total number of active nosepoke responses across nosepoke response extinction sessions. **(D)** Active nosepoke responses during the last extinction session and the cue-induced reinstatement test. **(E)** Active nosepoke responses during the saline- or heroin-priming reinstatement test. *n* = 9 rats per group. Data are means ± SEM. Ext, extinction; Reinst, reinstatement.

During Experiment 4, the experimental protocol was the same as in Experiment 1 except that the rats were subjected to the infusion of 5-AZA or vehicle intra-BLA bilaterally 6 h after the 15-min retrieval (see Figure 4A).

### Statistical analysis

The results were reported as mean ± SEM and analyzed using the two-way/repeated measures ANOVAs in GraphPad, v.9.0. Each experiment had a between-subjects factor for the infusion treatment (5-AZA vs. vehicle) and a within-subjects factor for the test (last nosepoke extinction day vs. cue-induced reinstatement test or saline-priming reinstatement test vs. heroin-priming-induced reinstatement test) (see section “Results”). We used Tukey’s *post hoc* tests to analyze the two-way ANOVAs for specific pair-wise comparisons and examine any significant main effects or interactions (*p* < 0.05, two-tailed).

## Results

### Experiment 1

Immediate post-reactivation 5-AZA treatment intra-BLA reduced subsequent cue-induced and heroin-priming-induced reinstatement of heroin-seeking behavior.

Two groups of rats were used in experiment 1 to examine the effects of post-reactivation 5-AZA infusion intra-BLA on cue-induced and heroin-priming-induced reinstatement of heroin-seeking behavior (Figure 1A). According to the schematic representation of the BLA regions, all cannula placements were within the BLA boundaries (Figure 1B). Analysis of the behavioral data of a mixed two-way ANOVA with the training date as a within-subjects factor, and the treatment (5-AZA vs. vehicle) as a between-subjects factor showed that the rate of heroin self-administration did not differ between the rats in the 5-AZA group (*N* = 10) and the rats in the vehicle group (*N* = 10), indicated by the total number of heroin infusions [main effect of acquisition date: *F*_(9_, _162)_ = 16.55, *p* < 0.0001; infusion treatment: *F*_(1_, _18)_ = 0.4890, *p* = 0.4933; acquisition date × infusion treatment: *F*_(9_, _162)_ = 1.556, *p* = 0.1327; Figure 1C]. Furthermore, there was no difference between the 5-AZA group and the vehicle group in the nosepoke extinction session as shown in the total number of nosepokes [main effect of extinction date: *F*_(8_, _144)_ = 23.21, *p* < 0.0001; infusion treatment: *F*_(1_, _18)_ = 0.2118, *p* = 0.6508; extinction date × infusion treatment: *F*_(8_, _144)_ = 0.4913, *p* = 0.8609; Figure 1D].

We found that the active nosepokes of the 5-AZA group significantly differed from the vehicle group in both the cue-induced and heroin-priming-induced reinstatement tests. A two-way ANOVA with the treatment (5-AZA vs. vehicle) as the between-subjects factor and test day (last extinction day vs. cue reinstatement day) as the within-subjects factor revealed a main effect of treatment [*F*_(1_, _18)_ = 7.236, *p* = 0.0150], a main effect of test day [*F*_(1_, _18)_ = 40.91, *p* < 0.0001], and a significant treatment × test day interaction [*F*_(1_, _18)_ = 9.947, *p* = 0.0055] during the cue-induced reinstatement test. The *post hoc* analysis revealed that the number of active nose pokes of the 5-AZA group was significantly decreased compared with the vehicle group during the cue-induced reinstatement test (*p* < 0.01) (Figure 1E right column). Furthermore, a two-way ANOVA with treatment (5-AZA vs. vehicle) as the between-subjects factor and test day (last extinction day vs. cue reinstatement day) as the within-subjects factor revealed a main effect of treatment [*F*_(1_, _18)_ = 4.570, *p* = 0.0465], a main effect of test day [*F*_(1_, _18)_ = 35.33, *p* < 0.0001], and a significant treatment × nosepokes interaction [*F*_(1_, _18)_ = 4.675, *p* = 0.0443] during the heroin reinstatement test. The *post hoc* analysis showed that the number of active nosepokes in the 5-AZA group was significantly lower than the vehicle group during the heroin-induced reinstatement test (*p* < 0.01) (Figure 1F right column).

These findings suggest that inhibiting the activity of DNMT with infusions of 5-AZA in the BLA immediately following the heroin cue retrieval significantly reduced cue-induced and heroin-priming-induced heroin-seeking behavior.

### Experiment 2

Immediate post-reactivation 5-AZA treatment intra-BLA reduced subsequent cue-induced heroin seeking and the spontaneous recovery of heroin-seeking behavior.

In experiment 2, we aimed to test the effect of immediate post-reactivation 5-AZA treatment intra-BLA on cue-induced heroin seeking reinstatement and the long-term effect on heroin reward memory in two groups of rats (Figure 2A). No difference was observed in the total heroin infusion after achieving heroin self-administration between the rats infused with either 5-AZA (*N* = 8) or the vehicle (*N* = 8) [main effect of acquisition date: *F*_(9_, _126)_ = 22.31, *p* < 0.0001; infusion treatment: *F*_(1_, _14)_ = 2.671, *p* = 0.1245; acquisition date × infusion treatment: *F*_(9_, _126)_ = 0.8167, *p* = 0.6018; Figure 2B]. Likewise, no differences were found between groups in extinction training [main effect of extinction date: *F*_(8_, _112)_ = 23.59, *p* < 0.0001; infusion treatment: *F*_(1_, _14)_ = 0.08584, *p* = 0.7738; extinction date × infusion treatment: *F*_(8_, _112)_ = 0.4240, *p* = 0.9045; Figure 2C].

Similar to the results obtained in experiment 1, there was a significant difference in the active side nosepoke between the 5-AZA group and the vehicle group in the cue-induced reinstatement test [main effect of test: *F*_(1_, _14)_ = 19.98, *p* = 0.0005; infusion treatment: *F*_(1_, _14)_ = 6.843, *p* = 0.0203; test × infusion treatment: *F*_(1_, _14)_ = 7.339, *p* = 0.0170; Figure 2D]. The *post hoc* analysis showed that active nosepokes were significantly reduced in the 5-AZA group compared with the vehicle group in the cue-induced reinstatement test (*p* < 0.01) (Figure 2D right column). In addition, in the spontaneous recovery test, active nosepokes significantly differed between the 5-AZA group and the vehicle group [main effect of test: *F*_(1_, _14)_ = 63.00, *p* < 0.0001; infusion treatment: *F*_(1_, _14)_ = 8.866, *p* = 0.0100; test × infusion treatment: *F*_(1_, _14)_ = 16.31, *p* = 0.0012; Figure 2E]. The *post hoc* analysis revealed that drug-seeking in the 5-AZA group was significantly reduced compared to the vehicle group in the spontaneous recovery test (*p* < 0.01) (Figure 2E right column).

The findings of experiment 2 suggest that the immediate post-reactivation 5-AZA treatment intra-BLA reduced subsequent cue-induced heroin seeking reinstatement and this effect lasted for 28 days.

### Experiment 3

5-AZA infusion intra-BLA with no reactivation had no effect on subsequent cue-induced and heroin-priming-induced reinstatement of heroin-seeking behavior.

In experiment 3, we examined whether the effect of DNMT in the BLA on the reconsolidation of heroin reward memory depended on retrieval by using the 5-AZA group (*N* = 8) and vehicle group (*N* = 8) of rats. Following the acquisition session of heroin self-administration and extinction session which is the same as experiments 1, rats were infused with 5-AZA or the vehicle intra-BLA immediately after exposing to the training chamber for 15 min with no cue exposure (Figure 3A). We did not find differences in the acquisition session of heroin [main effect of acquisition date: *F*_(9_, _126)_ = 25.84, *p* < 0.0001; infusion treatment: *F*_(1_, _14)_ = 0.7240, *p* = 0.4091; acquisition date × infusion treatment: *F*_(9_, _126)_ = 0.6614, *p* = 0.7423; Figure 3B] or the extinction session [main effect of extinction date: *F*_(8_, _112)_ = 37.20, *p* < 0.0001; infusion treatment: *F*_(1_, _14)_ = 0.2503, *p* = 0.6246; extinction date × infusion treatment: *F*_(8_, _112)_ = 0.2094, *p* = 0.9887; Figure 3C] between the two groups of rats.

In cue-induced reinstatement test, we did not find a difference in active nosepokes between the groups [main effect of test: *F*_(1_, _14)_ = 43.38, *p* < 0.0001; infusion treatment: *F*_(1_, _14)_ = 0.2478, *p* = 0.6264; test × infusion treatment: *F*_(1_, _14)_ = 0.08683, *p* = 0.7726; Figure 3D]. Furthermore, in the priming-induced reinstatement test, the groups did not differ from each other in active nosepokes [main effect of test: *F*_(1_, _14)_ = 71.04, *p* < 0.0001; infusion treatment: *F*_(1_, _14)_ = 0.04099, *p* = 0.8425; test × infusion treatment: *F*_(1_, _14)_ = 0.002420, *p* = 0.9615; Figure 3E].

Thus, these results indicated that the 5-AZA treatment intra-BLA without reactivation had no effects on the subsequent cue-induced and heroin-priming-induced reinstatement of heroin-seeking behavior, indicating that the effect of DNMT in the BLA on the reconsolidation of heroin reward memory was reactivation-dependent.

### Experiment 4

Delayed 5-AZA treatment intra-BLA following reactivation had no effect on subsequent cue-induced and heroin-priming-induced reinstatement of heroin-seeking behavior.

Finally, in experiment 4, we investigated whether the role of 5-AZA treatment intra-BLA in the reconsolidation of heroin reward memory had a time window by using two groups of rats infused with either 5-AZA (*N* = 9) or vehicle (*N* = 9) (Figure 4A). In line with experiments 1–3, no difference was found in total heroin infusion between the two groups in the acquisition sessions [main effect of acquisition date: *F*_(9_, _144)_ = 35.09, *p* < 0.0001; infusion treatment: *F*_(1_, _16)_ = 0.4635, *p* = 0.5057; acquisition date × infusion treatment: *F*_(9_, _144)_ = 0.5624, *p* = 0.8260; Figure 4B]. Furthermore, no group difference in the extinction session was found for active nosepokes [main effect of extinction date: *F*_(8_, _128)_ = 38.71, *p* < 0.0001; infusion treatment: *F*_(1_, _16)_ = 0.03292, *p* = 0.8583; extinction date × infusion treatment: *F*_(8_, _128)_ = 0.6184, *p* = 0.7612; Figure 4C].

However, intra-BLA 5-AZA treatment 6 h after the reactivation did not affect the subsequent cue-induced reinstatement of heroin seeking behavior, indicated by the number of active nosepokes had no difference between groups [maineffect of test: *F*_(1, 16)_ = 48.29, *p* < 0.0001; infusion treatment: *F*_(1, 16)_ = 0.08935, *p* = 0.7689; extinction date × infusion treatment: *F*_(1, 16)_ = 0.01919, *p* = 0.8915; Figure 4D]; or the priming- induced reinstatement, as the number of active nosepokes had also no difference between groups [main effect of test: *F*_(1, 16)_ = 72.48, *p* < 0.0001; infusion treatment: *F*_(1, 16)_ = 0.1839, *p* = 0.6737; test × infusion treatment: *F*_(1, 16)_ = 0.2131, *p* = 0.6506; Figure 4E].

These experiments indicated that the role of 5-AZA on heroin-seeking behavior was time-specific, and inhibiting the activity of DNMT should be within 6 h after reactivation to suppress the heroin-seeking behavior.

## Discussion

Our study examined the role of DNA methyltransferase (DNMT) in the BLA on the reconsolidation of heroin reward memory. The main findings are as follows: (1) DNMT inhibition in the BLA immediately after light/tone cue reactivation reduces subsequent cue-induced and heroin-priming-induced reinstatement of heroin-seeking behavior; (2) a 5-AZA infusion in the BLA without reactivation has no effect on the subsequent cue-induced reinstatement of heroin-seeking behavior; (3) the inhibitory effect of a 5-AZA infusion in the BLA immediately after the reactivation session on heroin-seeking behavior lasts at least 28 days. These findings indicate that the activity of DNMT in the BLA is required for the reconsolidation of the heroin reward memory and inhibiting the DNMT in the BLA attenuates heroin-seeking behavior by disrupting the reconsolidation of heroin reward memory.

A relapse caused by persistent heroin reward memory is a major challenge to the therapy of heroin addiction (Kalivas and Volkow, 2005; Robbins et al., 2008; Economidou et al., 2011; Torregrossa et al., 2011; Ma et al., 2012; Ewing et al., 2021). Studies on drug addiction in both humans and animals have demonstrated that either pharmacological or non-pharmacological intervention in reconsolidation has a great potential to prevent relapse (Lee et al., 2005; Xue et al., 2012; Lin et al., 2014; Chen et al., 2021a; Zhang et al., 2021; Xie et al., 2022). In our present study, we find that DNMT inhibition in the BLA disrupts reconsolidation and attenuates the subsequent heroin-seeking behavior of heroin reward memory. Our results are consistent with our previous study, further confirming the role of DNMT in the reconsolidation of heroin reward memory, and indicate that DNMT inhibition during reconsolidation may be a general way to prevent relapse across drug classes (Shi et al., 2015). In addition, the present results verify two aspects of reconsolidation that have been reported by previous studies, namely the requirement of new protein synthesis during the process and the need to be maintained for approximately 6 h (Valjent et al., 2006; Li et al., 2010; Wu et al., 2011; Lin et al., 2014; Chen et al., 2021a; Zhang et al., 2021; Xie et al., 2022). Overall, we find that immediate post-reactivation intra-BLA DNMT inhibition reduces subsequent cue-induced and heroin-priming-induced reinstatement of heroin-seeking behavior by disrupting the reconsolidation of heroin reward memory and this inhibitory effect depends on the reactivation session and has a limited time window.

It has been reported that the memory reconsolidation process involves DNA transcription and *de novo* protein synthesis (Nader et al., 2000; de la Fuente et al., 2015). DNA transcription is regulated by the epigenetic mechanisms of chromatin restructuring and DNA methylation, which play critical roles in the reconsolidation of memory (Levenson and Sweatt, 2005; Duvarci et al., 2008; de la Fuente et al., 2015; Gonzalez et al., 2019; Liu et al., 2022). DNMT, a widely expressed DNA methylation enzyme in the mammalian adult nervous system (Feng et al., 2005; Rahn et al., 2013), suppresses the transcription process by catalyzing the methylation of cytosine residues in DNA, causing the chromatin structure to compact and abolishing the transcription factors binding to the specific site of DNA (Miller and Sweatt, 2007; Selvakumar et al., 2012; Lyko, 2018; Shi et al., 2021). Thus, DNMT is thought to disrupt reconsolidation by inhibiting the binding between transcription factors and DNA. In this way, the inhibiting activity of DNMT is argued to positively regulate the reconsolidation of memory (Maddox and Schafe, 2011; Selvakumar et al., 2012). However, some studies show that the inhibition of DNMT disrupts the consolidation and reconsolidation of memory. This is inconsistent with aforementioned positive effect of DNMT inhibiting activity on the reconsolidation of memory (Miller and Sweatt, 2007; Miller et al., 2008; Zhao et al., 2010; Maddox and Schafe, 2011; Monsey et al., 2011; Pearce et al., 2017). One of the reasons for this discrepancy may due to the fact that the DNA needs to be re-repressed for memory re-stabilization. Thus, if the activity of DNMT is inhibited, the re-repression of gene transcription will be blocked and the memory may remain in a labile state and be impaired easily (Miller and Sweatt, 2007; Miller et al., 2008; Zhao et al., 2010; Shi et al., 2015). Moreover, DNMT inhibition may influence other epigenetic mechanisms such as histone acetylation to regulate reconsolidation of memory (Maddox and Schafe, 2011). This may explain the aforementioned inconsistency. In addition, a similar study found that DNA methylation in the LA is required for the reconsolidation of fear memory (Maddox and Schafe, 2011). Our previous study also revealed that the activity of DNMT in the BLA is required in the reconsolidation of cocaine reward memory (Shi et al., 2015). Thus, the present study is consistent with the findings of recent studies that indicate the role of DNMT in the reconsolidation of drug memory (Shi et al., 2015; Brown and Feng, 2017; Cannella et al., 2018; Urb et al., 2020). However, our study also has some limitations such as the lack of molecular evidence to explain the alteration of the activity of DMNT during/after the manipulation in the study. More experiments are needed in the future that focus on the specific molecular alterations and related signaling pathways during/after retrieval of heroin reward memory. Furthermore, as the epigenetic mechanisms involving DNA methylation and histone acetylation are complex (Mahan et al., 2012), other potential epigenetic mechanisms of the DNMT in the reconsolidation of drug memory need to be investigated in the future (Jarome and Lubin, 2014).

In conclusion, our study demonstrated that DNA methylation regulates the reconsolidation of heroin reward memory in the BLA. Our study also highlights the significant effect of epigenetic regulation, specifically DNA methylation, in the reconsolidation of heroin reward memory. Our findings provide theoretical support for the molecular mechanisms of reconsolidation of drug memory in the BLA and the development of potential therapies for heroin addiction.

## Data availability statement

The raw data supporting the conclusions of this article will be made available by the authors, without undue reservation.

## Ethics statement

The animal study was reviewed and approved by the Local Committee on Animal Care and Use and Protection of the Hunan Normal University.

## Author contributions

SQ, CY, and YL: conceptualization. CS and SH: data curation. SQ and CS: writing of original draft preparation. CS and CY: review and editing. YL: funding acquisition. All authors contributed to the article and approved the submitted version.
